# Current Applications and Future Perspectives of Artificial Intelligence in Face-Driven Orthodontics: A Scoping Review

**DOI:** 10.3390/biomimetics11020146

**Published:** 2026-02-16

**Authors:** Barbora Heribanová, Katarína Janáková, Juraj Tomášik, Daniela Tichá, Štefan Harsányi, Andrej Thurzo

**Affiliations:** 1 Department of Orthodontics, Regenerative and Forensic Dentistry, Faculty of Medicine and KORFS, Comenius University in Bratislava, Dvorakovo Nabrezie 4, 81102 Bratislava, Slovakia; janakova23@uniba.sk (K.J.); tomasik7@uniba.sk (J.T.); ticha46@uniba.sk (D.T.); 2 Institute of Medical Biology, Genetics and Clinical Genetics, Faculty of Medicine, Comenius University in Bratislava, Sasinkova 4, 81108 Bratislava, Slovakia; stefan.harsanyi@fmed.uniba.sk

**Keywords:** AI, diagnosis, soft tissue analysis, aesthetics, individualized treatment planning, deep learning, machine learning

## Abstract

Artificial Intelligence (AI) has introduced transformative possibilities in orthodontics by enhancing diagnostic precision, treatment planning, and aesthetic outcomes. In face-driven orthodontics, treatment objectives extend beyond achieving proper occlusion to optimizing facial balance and harmony. With the growing patient demand for aesthetic improvements, AI technologies enable clinicians to integrate facial analysis and dynamic soft-tissue evaluation into personalized treatment approaches. Research in this scoping review analyzed current applications of AI in face-driven orthodontics, focusing on diagnosis, soft-tissue assessment, and individualized treatment planning. A comprehensive search was conducted in PubMed and Scopus for studies published between 2021 and 2025. The review followed the PRISMA-ScR guidelines. Of 54 initially identified studies, 24 met the inclusion criteria after title, abstract, and full-text screening. Extracted data were organized according to the main application areas of AI in face-driven orthodontics. Most studies focused on AI-assisted facial analysis, 3D reconstruction, and treatment simulation. Deep learning models demonstrated high performance in soft-tissue prediction, aesthetic evaluation, and diagnostic accuracy. However, heterogeneity in datasets, a lack of standardized validation protocols, limited external validation across included studies and limited clinical applicability were identified as key limitations. AI-based facial analysis supports a shift toward individualized, aesthetics-oriented orthodontic planning. Although current evidence highlights its potential for improving diagnostic precision and treatment outcomes, further validation through large-scale clinical studies is essential for broader implementation in everyday practice.

## 1. Introduction

Artificial Intelligence (AI) has rapidly evolved into a transformative force across medicine and dentistry, driving innovation in diagnosis, treatment planning, and personalized care. The aim of this scoping review is to explore current applications of Artificial Intelligence in face-driven orthodontics, focusing on diagnosis, treatment planning, and facial aesthetic outcomes in clinical practice. The aim is also to answer the question: What applications of AI are used in face-driven orthodontics and what is the use in orthodontic diagnosis and treatment planning? In addition, this review outlines the existing limitations of AI use in clinical orthodontics.

**Figure 1:**
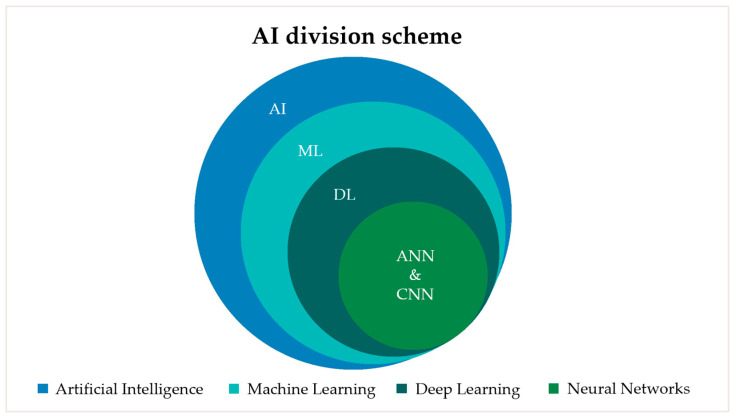
Graphic visualization of AI division scheme.

There have been significant advances in the application of Artificial Intelligence (AI) technologies. Machine learning (ML) is a branch of Artificial Intelligence that enables computer systems to learn from data and improve their performance on specific tasks without being explicitly programmed. Deep learning (DL) is a subset of machine learning that employs multi-layered artificial neural networks to automatically identify complex patterns within large datasets. The AI division scheme is shown in Figure 1. While traditional machine learning often relies on manually engineered features, deep learning models are capable of autonomously learning hierarchical representations directly from raw data. Deep learning includes Artificial Neural Networks (ANN) and Convolutional Neural Networks (CNN). Artificial Neural Networks (ANNs) are typically composed of fully connected (dense) layers. Convolutional Neural Networks (CNNs) incorporate convolutional layers for feature extraction, pooling layers for hierarchical spatial abstraction, and often conclude with dense layers to perform tasks such as classification or regression [1,2]. These advances have made AI-based systems powerful analytical tools in various areas of medicine, paving the way for their clinical translation in orthodontics and craniofacial research. Within orthodontics, a growing focus has been placed on aligning functional correction with overall facial harmony, giving rise to the concept of face-driven orthodontics [2].

Face-driven orthodontics focuses on matching proper occlusion with overall facial harmony [2]. It emphasizes the integration of attractiveness considerations into treatment planning, ensuring that orthodontic outcomes are consistent with the patient’s expectations for improved facial attractiveness [3]. Linking Artificial Intelligence to this approach increases diagnostic accuracy, personalizes treatment planning, and optimizes clinical procedures, thereby improving overall patient satisfaction and treatment outcomes [4,5,6]. Consequently, the combination of AI and face-driven orthodontics represents a paradigm shift from conventional occlusal-based treatment planning toward a holistic, patient-centered, and data-driven aesthetic analysis. Unlike traditional orthodontics, which primarily emphasizes dental alignment and skeletal relationships, face-driven approaches prioritize facial soft-tissue morphology and prediction, dynamic facial expressions, and patient-specific aesthetic goals as primary diagnostic and therapeutic determinants. Therefore, methodological differences include the integration of facial analysis, soft-tissue prediction, and outcome simulation into the diagnostic workflow, shifting clinical priorities toward individualized aesthetic optimization alongside functional stability.

As technological capabilities continue to expand, AI is expected to play an increasingly critical role in optimizing orthodontic workflows and improving aesthetic outcomes. As the field of orthodontics continues to advance, ongoing research and development of AI technologies will play a key role in addressing these challenges while fostering innovations that combine both function and beauty [7,8,9,10,11,12]. This review therefore aims to provide a comprehensive overview of current AI applications in face-driven orthodontics, identify existing limitations, and highlight potential directions for future research and clinical implementation.

From a biomimetics perspective, artificial intelligence represents a computational paradigm inspired by human cognitive and perceptual processes, particularly those of the brain and visual system. Machine learning and deep learning models emulate biological mechanisms such as neural signal processing, pattern recognition, learning from experience, and adaptive decision-making, which are fundamental to human facial perception and aesthetic judgment. In face-driven orthodontics, AI systems are trained to replicate expert-level clinical reasoning by integrating complex facial cues, soft-tissue morphology, and dynamic expressions into diagnostic and treatment-planning workflows. This biologically inspired modeling enables the simulation of human aesthetic assessment and predictive adaptation to individual facial characteristics, thereby aligning orthodontic decision-making with principles of biomimetic design. Consequently, AI-driven facial analysis and outcome prediction can be regarded as a form of digital biomimetics, in which computational models mimic human biological intelligence to support personalized, functionally stable, and aesthetically harmonious craniofacial treatment planning.

## 2. Materials and Methods

The studies included in this scoping review focused on the application of Artificial Intelligence technologies in face-driven orthodontic treatment. The review was conducted in accordance with the PRISMA-ScR guidelines, as confirmed by the completed checklist and flow diagram.

Search strategy

Relevant studies were retrieved from the PubMed and Scopus databases. The search was carried out on 1 February 2025 at 05:20 pm and included articles published between 2021 and 2025. The search terms included the keywords “orthodontics”, “face-driven”, “soft tissue”, “Artificial Intelligence”, and “AI”. Database-specific search strategies were applied: in PubMed, the search was conducted using Title/Abstract fields and relevant MeSH terms, with filters applied for publication years (2021–2025) and language (English); in Scopus, the search was performed using TITLE-ABS-KEY fields, with year and language limitations applied as stated in the inclusion criteria. The search query was as follows:

For PubMed:

(“Orthodontics”[MeSH] OR orthodontic*[tiab]) AND (face-driven[tiab] OR “soft tissue”[tiab]) AND (“Artificial Intelligence”[MeSH] OR artificial intelligence[tiab])

For Scopus:

TITLE-ABS-KEY (orthodontics OR orthodontic) AND TITLE-ABS-KEY

(“face-driven” OR “soft tissue” OR facial OR face) AND TITLE-ABS-KEY (“artificial intelligence” OR “AI”)

Study design

Inclusion criteria were: original research papers, reviews and comparative studies written in English language and published between 2021–2025. Only studies involving orthodontic patients or human facial data relevant to orthodontic diagnosis or treatment, studies describing, developing or validating AI-based models applied to facial analysis, soft-tissue evaluation, 3D facial reconstruction, aesthetic outcome evaluation or treatment simulation within face-driven orthodontics were included in the search.

Exclusion criteria included: studies published outside of the time frame 2021–2025 and written in a language other than English. Studies other than reviews and research papers, and not related to orthodontics or not involving facial or soft-tissue analysis were excluded from the search. Studies describing AI applications unrelated to diagnosis, treatment planning, or aesthetic evaluation (e.g., administrative or non-clinical applications), and studies lacking a clear description of an AI-based model or algorithm were also excluded from the search.

Initial screening was performed by reviewing titles and abstracts to identify potentially eligible studies. The research was performed at the same time jointly by two reviewers. There were no significant disagreements, everything was resolved through discussion and consensus, as the inclusion criteria clearly indicated the suitability of the selected articles, which was also agreed upon by both independent reviewers conducting the research. Based on the content of the included studies, focal thematic areas were identified and used to structure data extraction and synthesis. List of the variables extracted includes:

- type of AI model
- clinical usability
- data modality

Research question

The research question was formulated using the PCC model:

Population (P) = orthodontic patients

Concept (C) = AI models based on Artificial Intelligence in face-driven orthodontics

Context (C) = clinical application and research

Research question: What applications of Artificial Intelligence are used in face-driven orthodontics and what is the use in orthodontic diagnosis and treatment planning?

## 3. Results

**Figure 2:**
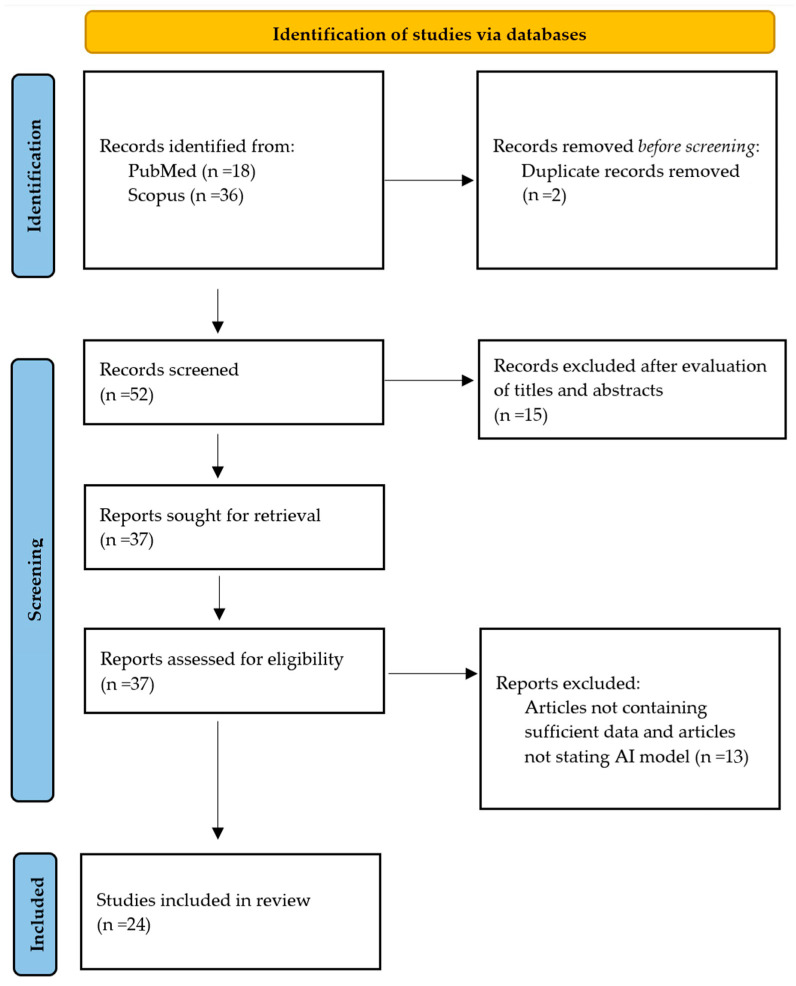
Selection of studies via PRISMA-ScR flow diagram.

After an initial search, 54 potentially relevant studies were identified. After removing duplicate records, 52 studies remained. Based on the title and abstract screening, 15 studies were excluded due to non-compliance with inclusion criteria. At the full-text review stage, 13 studies were excluded due to lack of orthodontic relevance, absence of facial or soft-tissue analysis, focus on non-clinical AI applications, or insufficient description of an AI-based diagnostic or treatment-planning model, were not written in English language or published between 2021 and 2025. The remaining 24 studies underwent assessment and met all predefined eligibility criteria. This scoping review was conducted in accordance with the PRISMA-ScR (Preferred Reporting Items for Systematic Reviews and Meta-Analyses extension for Scoping Reviews) checklist, as shown in Figure 2.

In total, 24 studies were subjected to full-text assessment when available and were included in the final review in Table 1, organized according to their primary application areas within face-driven orthodontics.

A simplified representation of the studies according to their use in clinical practice and type of AI model is shown in Table 2.

Within the diagnostic domain, included studies addressed two-dimensional (2D) facial analysis, three-dimensional (3D) facial symmetry and asymmetry assessment, and the diagnosis of facial dysmorphology. These investigations primarily relied on 2D facial photographs, 3D facial scans, and cone-beam computed tomography (CBCT) imaging data.

Within the landmark identification domain, studies focused on automated facial landmark detection, quantitative soft-tissue analysis, identification of skeletal abnormalities, automatic cephalometric analysis, and three-dimensional template-based determination of cephalometric landmarks. The datasets used in these studies consisted mainly of 2D facial photographs, lateral cephalograms, and CBCT images.

Within the treatment planning domain, included studies examined the prediction of lateral facial profile changes following orthodontic treatment, feasibility analyses of intelligent predictive models, prediction of extraction versus non-extraction treatment decisions, digital 3D smile design, evaluation of pre- and post-orthognathic surgical changes, visualization tools for predicting soft-tissue outcomes after orthognathic surgery, assessment of facial soft-tissue changes following bimaxillary orthognathic surgery in patients with cleft lip and palate, prediction of soft-tissue and alveolar bone changes after orthodontic treatment, and comparisons of facial growth prediction models. These studies utilized a wide range of imaging modalities, including lateral cephalograms, 2D facial photographs, 3D facial scans, four-dimensional (4D) video recordings, and CBCT images.

**Figure 3:**
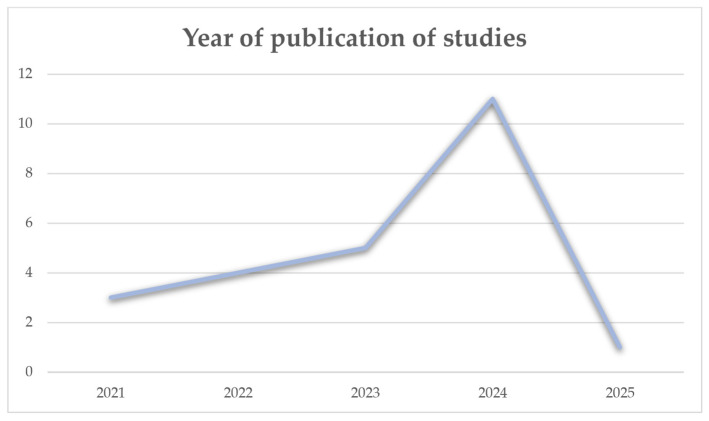
Graphic visualization of the rising trend of articles about the use of AI in face-driven orthodontics.

Each year the number of publications on the topic increases, which is represented by Figure 3, but as the review is conducted at the beginning of 2025, there has been only one study this year that has met the inclusion criteria so far. That is the reason for the decrease in the graph.

**Figure 4:**
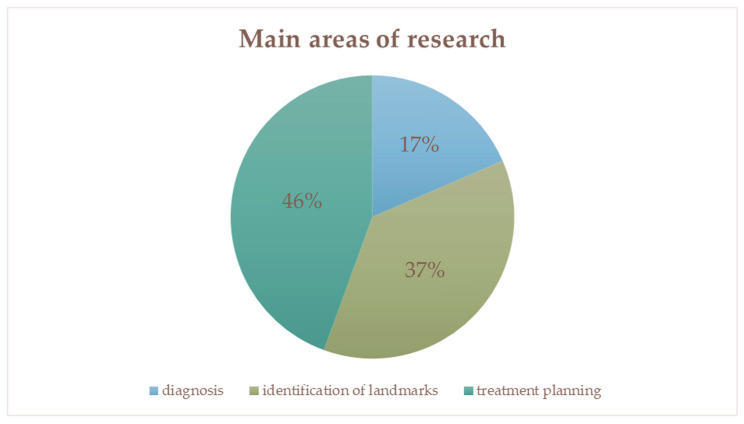
The divided main areas of research: Diagnosis 17% (*n* = 4), landmark identification 37% (*n* = 9), and treatment planning 46% (*n* = 11). The values given in the graph are percentages.

Figure 4 represents the distribution of studies by main area of research–diagnosis, identification of landmarks and treatment planning.

**Figure 5:**
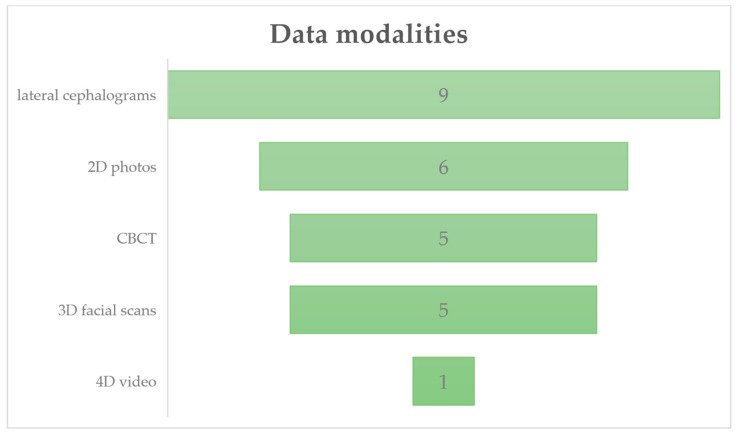
Division of studies according to the data modalities used in their research. Several studies used more than one data modality.

Figure 5 summarizes the distribution of the included studies based on imaging data utilized, encompassing 2D facial photographs, 3D facial scans, 4D dynamic video recordings, lateral cephalograms and CBCT imaging.

## 4. Discussion

Artificial Intelligence (AI) in face-driven orthodontics represents a significant advancement, emphasizing the interplay of harmonious outcomes and treatment efficacy. Aesthetics in dentistry has recently become a motivation for patients and often serves as the main reason for seeking out a specialist. Its importance is increasingly recognized in orthodontics, with studies showing that patients prioritize improved appearance when making treatment decisions. The aim of this scoping review was to explore current applications of Artificial Intelligence in face-driven orthodontics, focusing on diagnosis, treatment planning, and facial aesthetic outcomes in clinical practice.

Recent advances in Artificial Intelligence have enabled the processing of large volumes of imaging and clinical data with increasing precision, facilitating the integration of facial aesthetics into orthodontic diagnosis and treatment planning. The findings of this scoping review indicate that AI applications in face-driven orthodontics cluster primarily into three domains: treatment planning and outcome prediction, facial landmark detection and soft-tissue analysis, and automated diagnosis. Among these, treatment planning and outcome prediction emerged as the most extensively explored application, reflecting growing clinical interest in forecasting facial aesthetic changes and supporting goal-oriented orthodontic decision-making [5,37]. Across studies, AI-based landmark detection applied to lateral cephalograms, CBCT scans, 2D photographs and 3D facial scans demonstrated accuracy and precision comparable to expert clinicians, offering substantial gains in efficiency and reproducibility. However, common limitations that could affect the resulting performance could be different measurement methods, landmark definition variability, head posture standardization, and inter-device variability [38,39,40,41]. Machine learning and deep learning approaches, including artificial neural networks and convolutional neural networks, dominated this field, with some studies employing gradient boosting algorithms for structured datasets. While these models generally reported high performance, most relied on internal validation strategies, and external validation remained limited [42].

The studies suggest that intelligent diagnostics perform extremely well, with accuracy and precision similar to that of trained experts. Automation reduces the possibility of human error and enables the analysis of large data sets. These systems can simplify tasks and provide results quickly, which can save dentists time and help them perform their duties more efficiently [42,43]. The model of intelligent diagnosis methods may become increasingly accurate as data accumulates in the near future [6].

Artificial Intelligence can record facial features, enabling a comprehensive perception of the patient that provides additional information for treatment strategies [44]. The more the analysis of the face as a whole is taken into account, the more orthodontists will realize the importance of planning the ideal smile design from the beginning of treatment [12]. Artificial Intelligence technologies are being used to perform facial analyses that go beyond the simple assessment of proportionality and symmetry. These tools can identify facial characteristics that are essential for the development of customized treatment plans.

Harmonic results are most effectively assessed using complex three-dimensional (3D) analyses of facial structures that take into account image depth, different layers such as bone, muscle, fat tissue, and skin. This systematic approach allows professionals to select the optimal methods to improve facial appearance in orthodontic therapy. The creation of precise 3D models, for example, through facial scans, supports clinical decision-making so that the chosen treatment takes into account the individual patient’s facial proportions and meets their requirements about visual appearance [37,45].

Although this approach has several advantages over traditional manual procedures, its implementation in clinical practice is still limited, probably due to two main reasons: lack of technical expertise and the high costs associated with the equipment [46]. However, it is now possible to record a facial scan using a mobile phone or tablet [39,47,48].

In the recent past, the best practice was to use AI algorithms in cephalometric analysis, but the need to double-check the outcome data manually is still present [5]. A deep learning-based automatic soft tissue analysis model performs landmark detection and measurement calculations on, for example, orthodontic facial photographs, to achieve a more comprehensive quantitative soft tissue assessment. The system can automatically detect 43 landmarks on frontal images and 17 landmarks on lateral images of the face. The models can assist maxillofacial orthopedists in efficient and accurate quantitative soft tissue assessment in clinical practice. Between model prediction and manual measurements, there was no statistically significant difference [26]. The soft tissue profile of the face provides only limited information and is not sufficient by itself to thoroughly plan the final appearance of a smile customized to a given face [39].

Facial symmetry is increasingly important in today’s orthodontic treatment. However, the boundary of asymmetry is not clearly defined. Stereophotogrammetry has a distinct advantage in measuring facial asymmetry. Facial asymmetry can be quantified using three-dimensional technology, and it is also possible to investigate whether conventional assessment of facial asymmetry agrees with analysis using 3D technology, which has been reported to be reliable. By measuring the original face and its mirror image, it is possible to compare the correspondence of the surface of the individual regions, as well as the whole face, to calculate the degree of symmetry. Significant diagnostic values are in the area of the lips, chin, lateral parts of the mandible and cheeks. The most significant influence on facial symmetry has the mandible [42,49].

Video recordings of a patient talking and smiling can provide valuable information about the visual identity of the conversation and the dynamics of the smile that traditional still images do not provide [50]. Four-dimensional recordings are the basis for motion-simulating designs that overcome many limitations. However, it should be noted that this AI virtual smile design can sometimes be unrealistic and unattainable due to overlooking skeletal relationships, occlusion, and the shape of dental arches. Nevertheless, the use of new modern digital tools contributes to their updating and progressive increase in reliability [51,52,53]. By examining facial proportions and linear distances between areas, AI tools can provide insights that were previously difficult to quantify due to subjective assessment. These include assessments of facial proportions based on standard aesthetic models such as the golden ratio or Marquardt’s mask, although their generalizability remains limited [39]. This analytical ability also allows orthodontists to predict the need for tooth extraction or surgery. It is important to take into account the unique anatomy of an individual’s dentition to reflect the patient’s individual beauty, thereby providing a more holistic approach to orthodontic care [5,6,37].

A study revealed that the AI-enhanced images were perceived as more attractive, compared to the original real photographs, underscoring the effectiveness of AI in meeting aesthetic requirements. In addition, the use of AI tools allows that specific adjustments can be made based on individual patient preferences such as lip fullness and eye size, contributing to higher satisfaction rates [12,37]. AI also facilitates remote monitoring of patient‘s progress, improving adherence to the treatment plan and allowing for early interventions if necessary [52,54].

Artificial Intelligence has the potential to substantially advance face-driven orthodontics by enhancing diagnostic precision, treatment planning, and aesthetic outcome prediction. However, the ethical implications of AI implementation are particularly pronounced in this field due to the use of facial images and three- and four-dimensional facial data, which constitute highly sensitive biometric information [3,55,56].

Responsible integration of AI into orthodontic workflows requires clear governance frameworks addressing informed consent, secondary data use, secure data storage, and accountability for AI-assisted decisions [55]. Ethical oversight should be operationalized through human-in-the-loop models, ensuring that qualified orthodontists remain responsible for validating AI-generated outputs before clinical application [6,57,58,59].

## 5. Conclusions

Artificial Intelligence systems are becoming increasingly adept at simulating tooth movement and changes in the surrounding soft tissues. Such a simulation, or illustrative virtual reality, helps to improve understanding, motivation and, thus, patient involvement in the treatment process. Future developments may allow for real-time adjustments based on patient progress, optimizing treatment sequencing and potentially reducing the overall treatment duration [6,60]. Properly executed treatment achieves harmony between soft and hard tissues [50,60,61]. Several studies still undergo internal validation.

AI in face-driven orthodontics shows significant potential in enhancing diagnostic precision and aesthetic treatment outcomes, yet it must remain a tool under human clinical supervision [60,62].

Despite these advances, the integration of AI into face-driven orthodontics raises ethical considerations and challenges, particularly regarding the oversight of AI applications in patient care [55,56,63]. Also important is the need for appropriate communication among orthodontists to ensure a cohesive approach to treatment planning in which technology and patient-centered care are seamlessly integrated [54,64,65].

Despite the advances made, current AI technologies still have limitations that cannot be overlooked. Although AI can help significantly, it lacks the nuanced critical and emotional intelligence inherent in human clinicians. This limitation underscores the fact that AI cannot completely replace the expertise and judgment of experienced doctors, but clinicians who effectively integrate AI tools are likely to outperform those who do not adapt to digital advances [6,62,66,67].

## Figures and Tables

**Table 1:** Overview of included studies.

Reference	Title of the Study	Year of Publication	Type of AI Model	Use in Clinical Practice	Data Modality
[13]	Machine learning in orthodontics: Automated facial analysis of vertical dimension for increased precision and efficiency	2022	ML	2D facial analysis	2D photos
[14]	Revealing the representative facial traits of different sagittal skeletal types: decipher what artificial intelligence can see by Grad-CAM	2023	DL	identification of skeletal abnormality on the basis of soft tissues only	2D photos
[15]	Research of orthodontic soft tissue profile prediction based on conditional generative adversarial networks	2025	DL	predicting changes in lateral appearance after orthodontic treatment	lateral cephalograms
[16]	Future perspectives of digital twin technology in orthodontics	2024	ML	feasibility analysis of an intelligent predictive model	3D facial scans
[17]	Automatic three-dimensional facial symmetry reference plane construction based on facial planar reflective symmetry net	2024	DL	3D facial symmetry analysis	3D facial scans
[18]	A machine learning model for orthodontic extraction/non-extraction decision in a racially and ethnically diverse patient population	2023	ML	the ability to predict extraction/non-extraction decisions	lateral cephalograms
[19]	3D face mask for facial asymmetry diagnosis	2024	ML	3D asymmetry assessment	3D facial scans
[20]	Validation of ‘total face approach’ (TFA) three-dimensional cephalometry for the diagnosis of dentofacial dysmorphisms and correlation with clinical diagnosis	2024	ML	diagnosis of dysmorphia	CBCT
[21]	Smile Design: Mechanical Considerations	2022	ML	digital 3D smile design	2D photos & 3D facial scans & 4D video
[22]	Automated analysis of three-dimensional CBCT images taken in natural head position that combines facial profile processing and multiple deep-learning models	2022	DL	automatic cephalometric analysis	CBCT
[23]	Computerized three-dimensional cephalometric template for Thai adults	2023	ML	determination of cephalometric landmarks by creating 3D templates	CBCT
[24]	Automated facial landmark measurement using machine learning: A feasibility study	2024	ML	detection of facial landmarks	2D photos
[25]	Face comparison analysis of patients with orthognathic surgery treatment using cloud computing-based face recognition application programming interfaces	2023	DL	differences between before and after orthognathic surgery	2D photos
[26]	Automatic soft-tissue analysis on orthodontic frontal and lateral facial photographs based on deep learning	2024	DL	automatic soft tissue analysis	2D photos
[27]	Artificial intelligence for treatment planning and soft tissue outcome prediction of orthognathic treatment: A systematic review	2024	ML	visualization tool for predicting soft tissue outcomes after orthognathic treatment	CBCT
[28]	Three-Dimensional Facial Soft Tissue Changes After Orthognathic Surgery in Cleft Patients Using Artificial Intelligence-Assisted Landmark Autodigitization	2021	ML	facial soft tissue changes after bimaxillary orthognathic surgery in patients with cleft lip and palate	CBCT
[29]	Orthodontic treatment outcome predictive performance differences between artificial intelligence and conventional methods	2024	ML	prediction of soft tissue and alveolar bone changes after orthodontic treatment	lateral cephalograms
[30]	Reliability and accuracy of Artificial intelligence-based software for cephalometric diagnosis. A diagnostic study	2024	ML	automatic cephalometric analysis	lateral cephalograms
[31]	Is automatic cephalometric software using artificial intelligence better than orthodontist experts in landmark identification?	2023	ML	automatic cephalometric analysis	lateral cephalograms
[32]	Does artificial intelligence predict orthognathic surgical outcomes better than conventional linear regression methods?	2024	DL	predicting orthognathic surgery outcomes	lateral cephalograms
[33]	Comparison of individualized facial growth prediction models based on the partial least squares and artificial intelligence	2023	DL	comparison of facial growth prediction models	lateral cephalograms
[34]	Comparison of cephalometric measurements between conventional and automatic cephalometric analysis using convolutional neural network	2021	ML	automatic identification of anatomical landmarks	lateral cephalograms
[35]	Three-dimensional virtual planning in mandibular advancement surgery: soft tissue prediction based on deep learning	2021	DL	predicting the virtual soft tissue profile after mandibular surgery	3D facial scans
[36]	Accuracy of web-based automated versus digital manual cephalometric landmark identification	2024	DL	identification of cephalometric landmarks	lateral cephalograms

**Table 2:** Overview of included studies that have been divided into three groups based on the purpose of AI.

Use in Clinical Practice	Type of AI Model	References
Diagnosis	ML = 3, DL = 1	[13,17,19,20]
Identification of landmarks	ML = 5, DL = 4	[14,22,23,24,26,30,31,34,36]
Treatment planning	ML = 6, DL = 5	[15,16,18,21,25,27,28,29,32,33,35]

## Data Availability

No new data was created or analysed in this study. Data sharing is not applicable to this article.

## Abbreviations

The following abbreviations are used in this manuscript:

AI	Artificial Intelligence
ML	Machine learning
DL	Deep learning
ANN	Artificial neural networks
CNN	Convolutional neural networks
GB	Gradient boosting
3D	Three-dimensional
PCC	Population, Concept, Context
